# “They're causing more harm than good”: a qualitative study exploring racism in harm reduction through the experiences of racialized people who use drugs

**DOI:** 10.1186/s12954-022-00672-y

**Published:** 2022-08-25

**Authors:** Parnika Godkhindi, Lisa Nussey, Tim O’Shea

**Affiliations:** 1 University of Toronto, Toronto, Canada; 2 McMaster University, Hamilton, Canada; 3 Keeping Six - Hamilton Harm Reduction Action League, Hamilton, Canada

**Keywords:** Harm reduction, Racism, Drug policy crisis, Opioid crisis, War on drugs, People who use drugs, Policing in harm reduction, Qualitative research

## Abstract

**Background:**

Increased opioid-related morbidity and mortality in racialized communities has highlighted the intersectional nature of the drug policy crisis. Given the racist evolution of the war on drugs and the harm reduction (HR) movement, the aim of this study is to explore racism within harm reduction services through the perspectives of our participants.

**Methods:**

We conducted a qualitative descriptive study to explore the perspectives of racialized service users and providers on racism in the HR movement in the Greater Toronto and Hamilton Area (GTHA). Four racialized service users and four racialized service providers participated in semi-structured interviews that were audio-recorded, transcribed, and analysed thematically.

**Results:**

Five themes related to racism in HR were generated: (1) whiteness of harm reduction as a barrier to accessing services, (2) diversifying HR workers as a step towards overcoming distrust, (3) drop-in spaces specific to Black, Indigenous, and people of colour are facilitators to accessing harm reduction, (4) lack of representation in HR-related promotional and educational campaigns, and (5) HR as a frontier for policing.

**Conclusions:**

Our findings suggest that structural and institutional racism are prevalent in HR services within the GTHA, in the form of colour-blind policies and practices that fail to address the intersectional nature of the drug policy crisis. There is a need for local HR organizations to critically reflect and act on their practices and policies, working with communities to become more equitable, inclusive, and accessible spaces for all people who use drugs.

## Introduction

The opioid overdose crisis is a complex health and social phenomenon with devastating consequences for individuals, families, and entire communities. According to the Public Health Agency of Canada, rates of opioid-related morbidity and mortality have dramatically increased over the years, with opioid overdoses resulting in nearly 25 000 deaths across the country since 2016 [1]. Between 2005 and 2020, annual rates of emergency department (ED) visits in our community of Hamilton, Ontario, skyrocketed from 18.2 to 116.5 per 100 000; hospitalizations increased from 12.4 to 25.5 per 100 000; and opioid-related deaths more than quadrupled from 5 to 21.1 per 100 000. Moreover, the per capita statistics for ED visits, hospitalizations, and opioid-related deaths in Hamilton remain significantly higher than the 2020 provincial averages of 84.5, 13.7, and 16.4 per 100 000, respectively [2]. While the rising rate of opioid-related deaths in Hamilton has been an ongoing and significant public health issue for over a decade, the COVID-19 pandemic has exacerbated the crisis.

In a special report issued by the Ontario Drug Policy Research Network (ODPRN), researchers found that there have been more opioid-related deaths in “ethnically diverse” neighbourhoods during the COVID-19 pandemic [3]. These findings point to the intersectional nature of the drug policy crisis, whereby racialized individuals continue to experience drug use differently from white individuals [4]. The long history of the war on drugs, driven by xenophobia and discriminatory law enforcement practices, has led to a drug policy crisis with profound disparities in access to service and health and social outcomes for communities of colour in the USA and Canada [5, 6]. We use the term “drug policy crisis” to emphasize the systemic, political, and preventable nature of the devastation wrought upon communities by what is often called the opioid overdose crisis.

Rooted in grassroots activism, harm reduction (HR) is a means to reduce the harms associated with substance use, without requiring people to stop using drugs [7]. Its founding ideology focuses on structural change to address the social, economic, and racial inequalities faced by people who use drugs (PWUD). Yet with the recent adoption of HR by public health, the movement today has been reduced to a more apolitical, medicalized means of promoting health and mitigating individual harm [8]. While scientific literature has demonstrated that HR practices can effectively reduce the risks and deaths associated with illicit drug use, its implementation must be understood in a historical context of drug policy that has been largely a racist response to the group of people associated with drug use and not the dangers of the drug itself [7, 9, 10].

The racist nature of the war on drugs has been well documented and discussed in academic and community circles [11–14]. Further, racism in health care delivery and research is widely acknowledged and under-addressed [15–17]. However, few studies have researched the impact that racism has on access to HR services [18]. Given that substance use is prevalent among communities of all backgrounds, the purpose of this study is to examine how well HR spaces support the diversity of people who use drugs in Hamilton, with a focus on how racism manifests within the HR movement. Bearing in mind that the diverse experiences of racialized people cannot be thoroughly explored in a single study, it is our hope that this work can inform anti-racist practices in our own organizations. We further hope that this work can be a contribution to what we see as a necessary and lacking conversation in the HR community and spur others to take it up in their own work and organizations.

## Study setting and context

Hamilton is a city of 580 000 people in Southeastern Ontario, Canada. According to the 2016 Canadian census, approximately 1 in 5 people living in Hamilton identify as racialized [19]. There are several HR organizations operating in Hamilton which provide PWUD with unused needles/syringes, Naloxone, educational materials, safer sex supplies, peer support, and Consumption and Treatment Services (CTS). The authors of this study are members or supporters of Keeping Six—Hamilton Harm Reduction Action League (K6) in Hamilton, ON. K6 is a small grassroots organization led by people with lived experience of drug use whose mandate is to defend the rights, dignity, and humanity of PWUD.

At K6, we observed that our well-attended weekly drop-in sessions were being accessed primarily by a white population. Furthermore, two of the authors have participated in numerous stakeholder dialogues within the HR community and can attest that there is inattention to the impacts of racism in the drug policy crisis and a lack of diversity in upper management, despite many organizations purporting that they are committed to combatting racism. There are some organizations in the Greater Toronto and Hamilton Area (GTHA) doing the work of offering HR programming catered to racialized populations, but they remain few and far between. These observations compelled us to interrogate our own practices and those of other HR services in Hamilton. This study was originally undertaken as an undergraduate thesis at McMaster University, with the explicit aim of generating discussion and informing and improving our own practices at K6.

As a team consisting of one non-Black, non-Indigenous woman of colour and two white health workers with no lived experience of drug use, it is critical that we acknowledge the privileges inherent to our own intersectional identities. We are mindful of the fact that conducting a study to identify issues of differential treatment can further exacerbate the marginalization of racialized PWUD, as Hyett et al. (2019) have outlined in their work on deficit-based health research [20]. Specifically, research can be weaponized to further marginalization, as it is often used to place the onus on minoritized communities while obscuring the role of larger oppressive systems [21]. Our research is primarily informed by community members and scholars who are Black, Indigenous, and people of colour (BIPOC), though we underscore that the objective of this study is an interrogation of racism and not of “race”.

## Historical background

The war on drugs in Canada is rooted in racist economic and social policy. From the early twentieth century, the criminalization of drugs and alcohol was motivated by anti-Black, anti-Indigenous, and anti-Chinese racism, rather than to protect people from the bodily or social harms associated with the use of substances [22]. In 1884, an amendment to *The Indian Act* prohibited the sale of alcohol to Indigenous people as a means of policing and assimilating communities into the Canadian body politic [23]. With the passing of the *Opium Act* in 1908, Canada became one of the first countries in the Western world to introduce anti-drug legislation [24]. Opium smoking was largely associated with Chinese immigrant culture at the time, and Canadian officials used the opium ban to target Chinese Canadian communities and punish individuals who imported, manufactured, or sold opium for non-medical purposes [25]. In 1914, the *Harrison Narcotics Act* was passed in the USA to impose taxes on opium, coca leaf, and their derivatives [25]. Throughout North America and the British commonwealth, prohibition was spurred by fears of racial integration, Indigenous sovereignty, and the “non-British other” threatening white middle-class purity [10, 13, 26]. The development of punitive drug policies originated as a tool to repress racialized communities and further colonial dispossession.

The 1980s were characterized by a period of “moral panic” that linked crime, danger, and drugs such as crack and heroin to Black populations. President Ronald Reagan reaffirmed the US commitment to the “war on drugs” by increasing the law enforcement budget and mandatory minimum sentences for drug offenses [26]. Canada followed suit, and Prime Minister Brian Mulroney rolled out a “National Drug Strategy” with a series of legislations to increase street surveillance and militarize police forces from 1987 to 1999 [22, 27]. Since then, fear of drugs and crime has repeatedly been weaponized during election cycles to garner support for political platforms that prioritize “law and order” and “public safety” [22]. The Stephen Harper government brought about another era of punitive drug laws in Canada: by 2008, over three-quarters of the funding for the National Drug Strategy was being allocated to policing, at the expense of addictions treatment or mental health supports [28]. These policies were not evidence-based and resulted in significant health and social harms for PWUD and their communities [29].

Over the last three decades, the war on drugs in Canada has caused great harm, as government policies and priorities have subjected racialized populations to excessive and invasive policing [28]. The criminalization of drugs has been central to mass incarceration and state violence that disproportionately impacts Indigenous people and Black Canadians [26, 28, 30]. On the whole, estimates suggest that Indigenous people are overrepresented by over 600% in Canadian federal prison, while Black Canadians are overrepresented by 300% [28, 31]. White Canadians are underrepresented in the criminal justice system, though they are just as likely, if not more likely, to engage in “drug crime” [27, 32]. Canada does not collect and release systematic race-based data on the criminal justice system, making it difficult for researchers to fully gauge the disparate impact that the war on drugs has had on racialized communities [28, 33].

Faced with the devastating consequences of the war on drugs, PWUD, community members, and ultimately, healthcare providers, came together to propose a more compassionate, evidence-based approach to addiction: HR [26]. Spearheaded by LGBTQ community advocates, the first needle exchange programs in Canada opened in Vancouver, Toronto, and Montreal during the late 1980s to curb rates of HIV infection [34]. The availability of high-grade heroin had led to a surge in drug-related overdoses and HIV/AIDS infections in Vancouver’s Downtown Eastside [35, 36]. In response, activists and “guerilla groups” rallied to save lives by opening unsanctioned safe injection sites (SISs) in the city [12, 37]. North America’s first legal SIS, Insite, opened in Vancouver in 2003, offering a safe environment for people to inject drugs. Before long, SISs were established in Toronto, Montreal, and Ottawa. In Hamilton, the AIDS Network first initiated needle exchange programming in 1992 [38]. An overdose prevention site was established more recently in the city in 2018, with a second one opening in 2022 [37, 39, 40].

While these efforts to advance HR practices were impactful and heroic, it is telling that they were only taken up by mainstream public health organizations when over-prescription of opioid painkillers and addiction primarily impacted white communities in middle-class suburbs [41]. The opioid crisis of the 1990s was not the first of its kind: North America had already experienced a heroin epidemic in the 1960s and 1970s. However, this earlier epidemic had mostly impacted “inner-city” Black communities. Victims and their families were met with overwhelming contempt, rather than compassion [42]. Criticisms of the disease model of addiction notwithstanding, it was only when white populations became dependent on opioids that drug use started to be seen in the more sympathetic light of a biomedical disease, rather than a moral failing [28, 43, 44].

Finally, analysing media as a means of gauging public opinion and policy priorities, scholars have documented divergent attitudes towards white and racialized opioid users [45]. The media continues to highlight stories of the epidemic’s suburban white victims and emphasize their humanity [45]. A media analysis by Nederland et al. found that drug use in racialized communities is “not considered newsworthy”, whereas drug use in white, suburban neighbourhoods is commonly portrayed as being “tragic”, “surprising”, and “novel”, thereby contributing to a “racially coded” narrative [45]. The result is that white victims of the drug policy crisis have been centred in public discourse, while racialized victims have been erased [42]. Just as in the criminal justice system, Canada does not collect race-based statistics pertaining to opioid-related deaths. Absence of data has made it more challenging for researchers to identify disparities in opioid-related mortality among racialized communities [46].

In neglecting racialized populations within the current drug policy crisis, while simultaneously portraying it as a novel drug epidemic, the media has erased the “past and present realities” of Black communities in public discourse [42]. Furthermore, by eclipsing the impacts of the opioid epidemic on racialized populations, “colour-blind ideology” has resulted in a collective inability to directly address racism and inequities [45]. Though Black and Indigenous HR advocates have been experiencing and calling attention to these issues for years, they have often been denied the platform to reach larger audiences and researchers [47].

As the societal response to the drug policy crisis shifts towards one of HR as a matter of public health, it cannot take for granted that this shift will be applied to or benefit all equally. Service providers and organizations must keep in focus the historical and contemporary racism that is widespread in our health systems [16, 17, 48, 49]. Informed by knowledge of the war on drugs and the racist evolution of public health-driven HR, our research sought to reflect on racism within our own practices at K6. Our research was guided by the following questions: *What are ways in which racism is manifest in the HR movement in the GTHA? How does racism impact the accessibility of HR services to racialized people in the GTHA?*

## Study methods

The study employed a qualitative descriptive methodology, per Sandelowski, to explore the perspectives of racialized PWUD and service providers regarding HR services within the GTHA [50, 51]. The study was approved by the Hamilton Integrated Research Ethics Board.

At the time of the study, PG was an undergraduate Arts and Science student at McMaster University and a member of K6. PG proposed the study to K6 as her undergraduate thesis. She conducted all of the interviews and led the data analysis and report writing. TO was an infectious disease specialist with expertise in addiction medicine and low-barrier care for people who use drugs. He was a member of K6. He was involved with the conception of the study, recruitment, and report writing. He also participated in data analysis (thematic refinement). LN was a registered midwife completing her MSc in Health Research Methods. She was a founding member of K6 and its co-coordinator. She was involved with the conception of the study, the data analysis at all stages, and report writing.

### Sampling and recruitment

Recruitment of service providers followed a purposive sampling method, in which the study team compiled and contacted a list of candidates based on their professional expertise and connections to the PWUD community. Service users were recruited by healthcare providers and peers, as well as via advertisements in HR/community health agencies. Service users were also recruited through snowball sampling, whereby one participant would recommend another prospective participant in the community. Inclusion criteria included any self-identified English-speaking racialized person (Black, Indigenous, or person of colour) in the GTHA who used HR services or worked as a HR service provider, aged 16 and older.

### Data collection and analysis

Data were collected for the study through semi-structured interviews. All participants provided informed written or verbal consent prior to the interview. Interviews lasted between 60 and 90 min. Participants were asked a variety of questions regarding the level of diversity in HR, barriers to access, and policing. These questions were informed by literature on racism in HR, insights from K6 members, and observations from the interviews themselves. (The service providers were conducted first and informed the development of the interview guide for the service users.) Financial incentives were not offered to the service providers, while a cash compensation of $20 was offered to participants who were service users. Interviews were conducted and recorded over Zoom. The video recordings were transcribed within 48 h of the interview, at which point the data were anonymized and deidentified of any personal or identifying information.

Interview transcripts were managed and organized using NVivo, a qualitative data management program. Data were analysed using inductive thematic analysis, an iterative method comprising six steps, as outlined by Braun and Clarke [52]. Our analysis was grounded in critical race theory concepts of institutional and structural racism to reflect on how race, class, and other systems of power impact individual experiences of HR [53]. We also used Kimberle Crenshaw’s theory of intersectionality as a framework for analysis, to “map the margins” and examine how the experiences of racialized PWUD may be located within structures of power [54, 55].

## Results and analysis

Four interviews were conducted with racialized service providers, and four interviews were conducted with racialized service users. In total, five themes were generated. The first theme was that whiteness in HR spaces can pose a barrier for racialized PWUD. The second theme pertained to diversifying HR workers as a step towards overcoming distrust. Thirdly, virtual and in-person drop-in spaces specific to BIPOC are facilitators to accessing HR. A fourth theme pertained to a lack of representation in HR-related promotional and educational campaigns. Finally, the fifth theme refers to the role of policing in HR.

### Whiteness of harm reduction as a barrier to accessing services

Most participants spoke of how HR spaces tended to be accessed primarily by white service users, with a noticeable absence of racialized communities. Several participants shared that drug use was a sensitive issue within their culture, where there is a lot of “*shame*” surrounding addiction, mental illness, and HR services. For these reasons, one participant commented, racialized PWUD often *“don’t even bother seeking those services out. And then when they do seek them out, if they’re […] all white, it can be discouraging”* (P2, service user).

In addition, participants commented that HR organizations are predominantly led and staffed by white people. One service provider shared that at all levels, upper management within HR was overwhelmingly white, commenting that:

It’s a very much white-dominated space. From the director of the [provincial network], to people coordinating and doing the engagement work, they’re not representative of the diversity in this province. (P6, service provider)

Many added that the lack of diversity in upper management can make racialized HR staff feel unwelcome, with one service provider sharing that they had encountered resistance when trying to introduce a BIPOC-specific day at their organization:

It sent a very clear message that […] ‘this is my space, and I am offering you a little moment here, so take it as it comes.’ (P7, service provider)

Another participant expressed frustration that programming for racialized people is encouraged only under very specific circumstances, like Black History Month. As a result, racialized HR workers often feel tokenized within the HR movement, as their suggestions and needs are frequently dismissed:

I'm often tokenized: ‘Can you come in and talk about the Black experience?’ […] While they say it's a non-judgmental environment, it still is, because it's very exclusionary, it's still very much a white space. And it's only necessary for Black workers to come in if it's, you know, Black History Month and we need to do an Indigenous, Black, and South Asian training module. […] When they're responsible for doing one culturally specific training, that's when they know us, but any other time, HR doesn't know Black workers, period. (P6, service provider)

In addition to a lack of diversity and tokenization, participants also described how whiteness manifested as overt and explicit racism in HR spaces through the use of hateful language and stereotyping by white service users. One participant recounted how they often heard people using *“racist slurs like [the N-word]”* (P4, service user).

The lack of diversity among service users and providers, compounded by overt racism, clearly deterred racialized people from entering HR spaces. One service provider spoke to the catch-22 of trying to provide life-saving services in a racist environment, and the impossibility of diversifying to better meet the needs of racialized PWUD:

I'm very protective of what spaces I’m bringing folks into, especially when I’m saying, ‘Hey come with your lived experience, we need you,’ but then the space isn't safe for them. It’s not even safe for me! P7 (service provider)

Participants consistently reported that HR spaces were, in general, inhospitable, and even harmful to them as racialized people. Participants spoke of a longing for spaces that were familiar, welcoming, and met their needs.

### Diversifying HR workers as step towards overcoming distrust

All participants emphasized that racialized service users have unique needs that are best addressed by racialized staff. They highlighted the urgent need for HR organizations to hire more racialized peer support workers, particularly given the mistrust that historically excluded communities might harbour for public institutions. For instance, one participant pointed out that there was a tenuous relationship between their community and HR:

When we look at the Black community, I can’t name one institution we trust, and it’s based on our experiences. Not because we're coming with this prejudice and bias and not giving it a chance—we gave it a chance, we tried accessing a service there, and look what happened. (P7, service provider)

In addition to distrust towards institutions, broadly speaking, participants mentioned that they had difficulty connecting with service providers who couldn’t relate to the way stigma and “*familial shame”* surrounding drug use operated in their own backgrounds.

Whereas there might not be as crazy of a stigma in a North American culture, in [my] culture, or a very religious culture, it’s like the end of the world […] and it’s hard to express that to a Westerner. (P3, service user)

Given the extent of mistrust and disconnect, some participants felt that organizations would benefit from having racialized HR workers who could “*be unique in our approaches”* and help *“establish trust, especially in a community that doesn't feel supported by social services”.* Participants felt that racialized staff may be able to provide more culturally sensitive support in a wider range of languages. One participant recounted an instance when seeing a caregiver from a similar cultural background had been particularly therapeutic for them:

I required the least amount of […] therapy and other resources, specifically because I could just talk to her about things that she would get. […] Visiting her took the place of other support services that I required, because she just did such an effective job of making me feel cared [for]. (P2, service user)

Participants felt that increasing the diversity of HR staff, such that service users could see themselves reflected in the organization, was an important step to address racism in HR spaces. They also described carving out BIPOC-specific service user spaces as a necessary measure.

### BIPOC-specific spaces (virtual and drop-in) are facilitators to HR

Many of the participants shared numerous reasons that physical and digital drop-in spaces for BIPOC were essential to uphold the needs and safety of racialized PWUD. On the one hand, drop-in spaces exclusive to BIPOC could help to protect and keep track of transient, at-risk community members, who may otherwise be difficult to reach:

We have a lot of murders of Indigenous women and stuff like that that need to be solved […]. I think what those spaces do is give us a way to keep tabs on those at-risk communities. And we get a sense of who they are as individuals, a little bit more, so that we can keep better check on them. (P1, service user)

Moreover, drop-in spaces exclusive to racialized people could help service users find social support within their communities, by hearing and learning from those who may have a similar cultural background. Participants highlighted the value in connecting people with a common heritage and shared experiences, as it was inspiring and reaffirming to see how other racialized people were navigating the hurdles in their own lives. For instance, one participant shared that drop-in spaces exclusive to racialized people could help promote *“person of colour solidarity”* and offer critical *“perspective, which was a huge part of the recovery”* (P2, service user).

Some participants expressed that physically entering HR spaces could be difficult for potential service users for a number of reasons. For example, one participant discussed how accessing HR may cause anxiety for some racialized service users due to a fear of being identified in small communities. The participant highlighted how their own HR team had introduced online drop-in spaces exclusively for racialized youth during the COVID-19 pandemic, to *“connect with folks where they are”* (P5, service provider). They went on to say that a “*drop-in online could be a space where people are able to show themselves and be themselves, where they don't have to put on a play for other people.”* Overall, online spaces were viewed by some participants as a lower-risk, lower-barrier way of engaging with HR.

### Lack of representation in HR-related promotional and educational campaigns

There was consensus that HR organizations rarely display diverse faces or perspectives on their promotional materials. Newcomer participants remarked that advertising is not always placed in locations frequently accessed by immigrant populations. To increase outreach, some participants recommended advertising in places frequented by diverse communities; suggestions included the *“Mandarin Hour on radio”* or the *“Indian grocery store”.* One participant felt that translating promotional materials would help, as *“being able to communicate effectively in a multitude of languages is really important, because we want to eliminate that communication barrier”* (P1, service user).

Most participants commented that promotional campaigns can play a significant role in reaching diverse populations. They shared that racialized PWUD might encounter challenges when discussing drug use and HR with their families, largely due to cultural stigma and unfavourable perceptions of substance use and mental illness. In this familial context, participants felt that cultural, racial, and socioeconomic diversity were especially key when advertising HR:

It’s important to have that cultural diversity, so people see that [drug use] can hit anybody and see how non-discriminatory it really is. (P1, service user)

One participant noted that diverse representation in promotional campaigns could help overcome cultural stigma and assist racialized PWUD in seeking support from their own families and broader communities:

The problem is that in these communities, a lot of times, we're just ignoring [drug use] completely and pretending like it's not there. That’s the biggest hurdle to why people like me can’t even bring it up with their family. […] But for parents to see someone who looks like their son, and he’s got a drug problem, he’s got a needle in his arm, he’s got marks up his hand, […] and then seeing that that person who looks like their son is accessing the services he needs and has gotten better. Then they can themselves accept that. (P2, service user)

Participants spoke to the ways whiteness in HR spaces impeded their participation and that diversifying staff and carving out space for racialized service users could help to facilitate access to meaningful service. This could not be accomplished, however, without specific attention to the impact of policing on access to HR.

### HR as a frontier for policing

All the participants commented on the disproportionate impact that policing has on racialized PWUD. Some participants expressed concerns that HR organizations have become *“fishing areas”* for the police, who often wait around to harass or detain service users. Due to heightened police presence around HR organizations, several participants expressed that they felt uncomfortable accessing critical services. Moreover, participants noted that the police would routinely single out and target racialized people.

One participant shared a model of negotiating with the police to protect BIPOC service users. As a service provider, they had observed how the police would wait at their organization’s doors to *“arrest and harass”* people accessing HR services. Seeing the harm that these interactions were causing to the community, P5 took up the task of negotiating with police to uphold *“the safety of communities of color, more specifically, the safety and well being of Black men”.* P5 outlined how their organization had successfully made a concerted effort to communicate with police officers over the past few years and felt that this method could be applied in other municipalities, as well:

We continue to make the calls, we continue to inform elected officials and police boards we otherwise don’t like, to say, look, police don’t need to focus their activity here, because there are community-based organizations taking care of the folks that you’re over policing. We don’t necessarily like each other, but there is an understanding that these conversations are for the greater good. […] This can certainly be replicated in other areas and regions. (P5, service provider)

The deterring and damaging impact of policing in general is compounded by racist policing practices and significantly deters racialized PWUD from accessing HR services. As one participant put it, *“white people are getting arrested, so I don’t stand a chance!”* (P1, service user).

## Discussion

Overall, the interview findings indicate that racism permeates the HR movement in the GTHA. Critical race theorists have proposed the term “structural racism” to describe how interconnected institutions like housing, health care, political participation, and the criminal justice system mutually reinforce inequitable practices and outcomes within one another [56]. Structural racism is not something that a select few individuals or institutions choose to enact, but rather an everyday feature of our social, political, and economic systems [57, 58]. Furthermore, “institutional racism” refers to racism that is embedded and normalized within laws, policies, and practices of societal institutions [59].

Similar to many health services, HR organizations rarely explicitly discriminate against racialized people; indeed, most of these organizations espouse very progressive ideals. However, they have largely adopted colour-blind practices with the stated intention of treating all people the exact same, in the face of systemic racism [60]. Scholars and community advocates have illustrated that such “post-racial” ideology exacerbates racial inequality by denying its very existence [61, 62]. In that regard, Hughes et al. have articulated the need to dismantle Euro-centric, hierarchical models of HR in favour of community-based approaches drawing from the experiential knowledge of racialized service providers [63]. Without focused efforts to dismantle power structures and injustice, HR organizations are bound to uphold structural and institutional racism through their policies and practices.

Our study adds to a hitherto limited discussion on the existence of racism within HR and explores some ways forward from the point of view of our participants. Our findings align with existing literature on cultural attitudes towards drug use, racial matching in health and social services, and racism in the criminal justice system.

It is well documented in addictions research that cultural views can impact help-seeking behaviours among immigrant populations—stigma can lead to racialized people concealing their drug use, instead of seeking support for it [64, 65]. Our findings reveal that the whiteness of HR spaces compounds the reluctance on the part of some racialized communities or individuals to seek out support. Rather than pathologizing and deriding communities of colour, as is commonly done in health service research, our findings point to the need for HR organizations to adopt more innovative approaches to better reach and welcome racialized PWUD.

Many studies in psychology have explored the advantages of being cared for by someone from the same cultural background. One meta-analysis found that individuals have a more positive perception towards therapists of their own race and ethnicity, as clients tend to trust and relate better with counsellors that share their cultural heritage [66]. A smaller number of studies examining “racial matching” in HR and addiction medicine suggest that increased representation has a positive impact on service engagement and treatment outcomes, as racialized service providers may better understand the needs of their communities [63, 67]. The results of our study, consistent with the literature, speak to how the whiteness of HR services is impacting the potential for racialized service users to benefit from these same services.

Finally, many participants noted that police presence near HR services had deterred them from accessing important supports. It is well known that law enforcement and criminalization of drug use disproportionately impact racialized PWUD, leading to overrepresentation of Black and Indigenous people within carceral spaces [22, 28]. However, there is a paucity of research on the intersection of HR, racism, and policing. Notably, two companion articles from 2020–2021 explored Toronto Police Service (TPS) officers and Consumption and Treatment Services (CTS) users/providers experiences of one another. Police expressed that they lacked clarity about how and when to engage with the CTS, as well as frustration at the reluctance of service providers to collaborate with them [68]. The exploration of service provider and user experiences of policing calls for a “bounding” of police authority; communication between service provider and policing leadership; and explicit written policy on how, when, and why police can enter the CTS [69]. Astonishingly, though perhaps tellingly, neither of these recent studies mentioned race or racism beyond stating the demographic makeup of the participants.

Our study has several limitations. For one, it has a small sample size of eight participants. The results would potentially be more conclusive and insightful if a greater number of racialized PWUD in the GTHA had been interviewed. In addition, we only interviewed English-speaking service users and service providers, meaning we were not able to account for the perspectives of non-English speakers. Because of our small sample size, there was no possibility to disaggregate our findings by racialized group; still, it is important to avoid assuming that the experiences of racialized groups are homogeneous. We also recognize that our own positionality as researchers may have impacted what participants were willing to share. The interviews were conducted by a racialized woman, but the study was driven by academics and health providers with no lived experience of drug use. Finally, because this study was taken up as a time-limited undergraduate thesis, we did not explicitly involve PWUD in steering the research. Despite the lack of involvement of PWUD, we felt compelled to publish this work because of the dearth of conversation in the literature regarding racism in HR.

It is clear from our findings that there is enormous room for HR organizations to make changes to their policies and practices to improve accessibility to BIPOC populations. While suggestions to that end can be elicited from our findings (increasing diversity of staff and management, better representation in promotional material, and implementing measures to address the deterrent and punitive impacts of police on service seekers), we implore HR organizations to take action in ways that will not further marginalize and exploit racialized people. What does it mean for the very institutions that have given rise to racist environments to be the ones responsible for implementing change? In acknowledging the racist origins of first the drug war, and now the HR movement, it is imperative that organizations take up a political analysis that does not simply arc towards “inclusive” practices that reproduce the problem by tokenizing, overburdening, and directly harming racialized people. Echoing the calls of Hughes et al. for a community engaged approach to HR, we suggest that funders and policymakers alike make room for more HR organizations and services that are led by BIPOC from their inception [63].

The theme of HR as a frontier for policing has important implications for practice. Our findings point to the need for HR providers to be cognizant of the impact policing is having on how racialized people can engage with services. We suggest organizations should be prepared, proactive, and transparent regarding how they plan to engage with police to protect service users and providers. It is important to acknowledge and plan in the context of calls to defund or abolish the police, which have continued to grow as a counter to the racist, colonial past and present of the policing institution [22].

In keeping with the limitations of the size and scope of this study, we cannot offer prescriptive, generalized recommendations to all HR organizations broadly. Rather, we encourage HR services to consult with community and take up this work in their own context. The findings from this study and our engagement with racialized service users and providers have prompted us to take up some concrete measures. In the immediate short term, K6 has or will:

- Engage its leadership and core membership in cultural safety and anti-racism training, including the history of racism in the war on drugs and health care.
- Build the capacity of the K6 leadership and membership to intervene and address racist harm between participants accessing our services.
- Work with a partner organization to hire and support racialized peer facilitators who can develop HR programming in consultation with racialized PWUD, including consideration of a BIPOC-specific drop-in.
- Liaise with police in a manner that is loyal to K6 members and supporters in the context of the growing movement to defund the police.

For us at K6, this study was a part of a first step in developing anti-racist practices—recognizing the problem of racism, asking hard questions, and reflecting on the answers without defensiveness. In keeping with the HR ethos of “nothing about us, without us”, we are endeavouring to develop relationships with organizations of and for racialized people in the city [70]. This is enabling us to share our expertise in HR practices, while fostering relationships of trust that will ultimately lead to the betterment of our work to defend the rights, dignity, and humanity of all people who use drugs.

Finally, as Hughes et al. remind us, HR services must go beyond addressing racism within our institutions, to externalizing and operationalizing the fight against racial, social, and economic injustices that give rise to the health inequities and barriers affecting PWUD [63]. It is critical to stay true to HR’s roots as a movement for broader structural change [8].

## Conclusion

This study clearly affirms for us as HR advocates that there is a long way to go in making HR services accessible to racialized people in the GTHA. The findings suggest that structural and institutional racism is prevalent in Hamilton area HR services, in the form of colour-blind policies and practices that fail to address the intersectional nature of the drug policy crisis.

While there is extensive literature on the presence of racism within the war on drugs and health care, ours is the first qualitative study to examine racism as it manifests within HR services. This study provides an important contribution to the field by examining the perspectives and experiences of racialized service users and service providers. It identifies potential areas for improvement and offers points of reflection for HR organizations.

HR organizations in the GHTA must live up to the movement’s principles of “nothing about us, without us” [70]. It is time to listen to what racialized people in and around the HR movement have long been saying: racialized PWUD will continue to be pushed through the cracks unless and until we gather the courage to have honest and critical conversations on racism in HR. We must also act on those conversations with integrity. Developing an anti-racist framework for HR will require urgent and collaborative efforts from institutions and community members alike and is a needed step to meaningfully and impactfully address and redress health disparities for racialized PWUD.
